# MeshCut data augmentation for deep learning in computer vision

**DOI:** 10.1371/journal.pone.0243613

**Published:** 2020-12-23

**Authors:** Wei Jiang, Kai Zhang, Nan Wang, Miao Yu

**Affiliations:** 1 School of Mechanical Engineering, Sichuan University, Chengdu, China; 2 School of Physical Science and Technology, Southwest Jiaotong University, Chengdu, China; Columbia University, UNITED STATES

## Abstract

To solve overfitting in machine learning, we propose a novel data augmentation method called MeshCut, which uses a mesh-like mask to segment the whole image to achieve more partial diversified information. In our experiments, this strategy outperformed the existing augmentation strategies and achieved state-of-the-art results in a variety of computer vision tasks. MeshCut is also an easy-to-implement strategy that can efficiently improve the performance of the existing convolutional neural network models by a good margin without careful hand-tuning. The performance of such a strategy can be further improved by incorporating it into other augmentation strategies, which can make MeshCut a promising baseline strategy for future data augmentation algorithms.

## Introduction

Recently, convolutional neural networks (CNNs) have demonstrated massive potential in the field of computer vision [1–12]. Modern CNNs commonly contain millions of parameters to try to achieve sufficient representational power for difficult tasks, but the excessive representational power inevitably risks overfitting, resulting in poor generalisation.

Data augmentation is an effective regularization strategy [13] by which more training samples can be generated to alleviate overfitting. Thereinto, geometric transforms and photometric distortions are two widely used methods. For geometric transforms, the basic operations include random scaling, flipping, rotating, and cropping. For photometric distortions, the operations include contrast, brightness, and hue. Although the two methods are pixel-level adjustments, the original information is left intact. Another type of data augmentation method simulates object occlusion, which can help the network learn relatively weak target features and improve the perception ability, thus increasing the network’s robustness. Typically, random erasing [14] and CutOut [15] randomly zero out one continuous block region in the input image. The HaS [16] and GridMask [13] methods randomly or evenly remove several block regions in an image. Expanding those concepts by applying them to feature maps are DropConnect [17], DropOut [18], and DropBlock [19]. Other proposed methods use data augmentation based on multisource image fusion; for example, MixUp [20] and CutMix [21]. Of these, MixUp superimposes two images with different pellucidity, and CutMix fills a cropped image with the rectangle regions of other images.

Among the abovementioned methods, the information occlusion methods, as one of the most promising methods, have demonstrated their specific ability to prevent the overfitting. However, there exist some deficiencies in practical use, such as the following: 1) The overfocused partial information dropping could not be inherently avoided, resulting in the incompatibility for small objectives. 2) Little attention was paid to the occlusion of context information, leading to the weak ability to learn the partial features. 3) The information confusion could not be effectively simulated, which was disadvantageous for further improving the robustness.

In the recent studies, some CNNs [22, 23], in combination with the recurrent neural network (RNN), have been used in an attempt to capture more target features that are inherent in a video sequence. These excellent methods provide a multi-state representation for the target and module the uncertain transformation for the background; hence, their performance in terms of accuracy is improved tremendously. Inspired by these ideas, we are intrigued to observe that global information fragmentisation has some similar effects, which is helpful for solving the three abovementioned problems. On this basis, we propose a novel augmentation method named MeshCut.

## MeshCut

**Fig 1:**
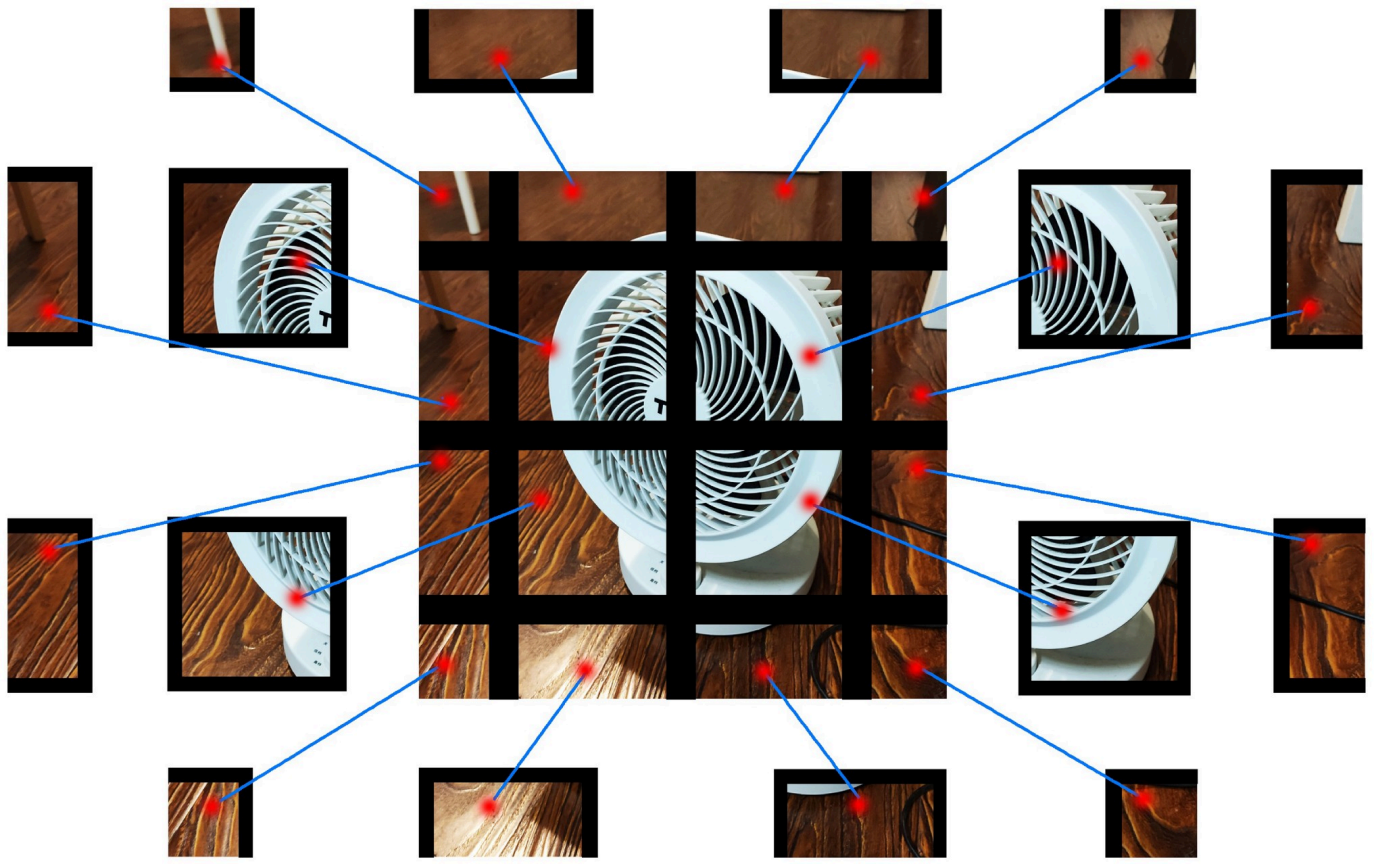


MeshCut is a simple, universal, and effective strategy that can be easily implemented in most of the existing CNN models. Different from previous methods, MeshCut, by superimposing a mesh mask in the training phase, transforms an image into a mosaic made with several image fragments, as shown Fig 1. Because of the zeroed-out margins between fragments, the overall feature of the target is effectively broken up into several partial features, which can provide more diversified information for the network.

**Fig 2:**
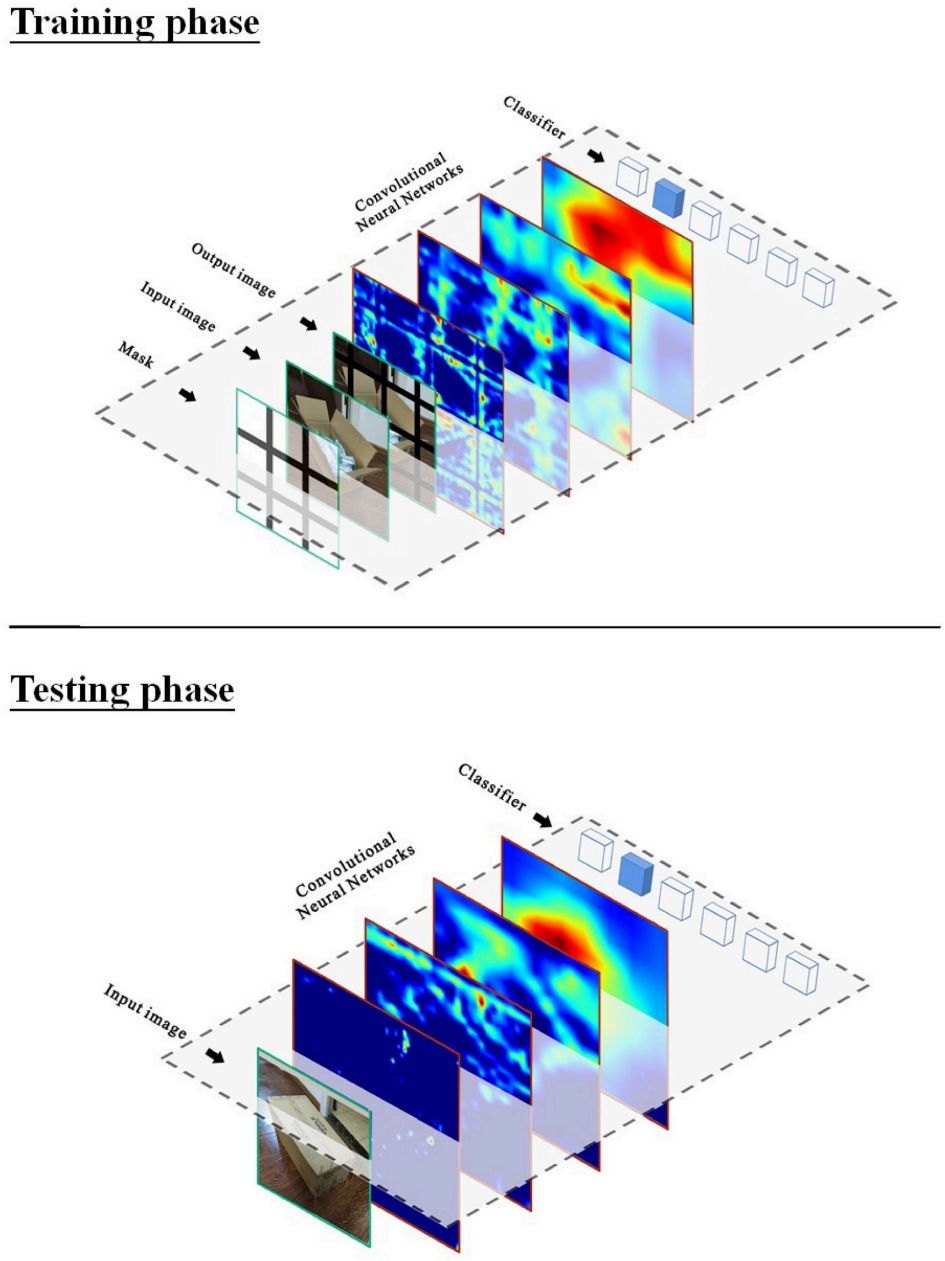


The MeshCut processing flow is shown in Fig 2. During the training phase, MeshCut overlays a binary mask M on the input image, namely

$$\begin{array}{c} I_{\text{out}}=I_{in}\times M, \end{array}$$ (1)

where *I*_*in*_ ∈ *R*^*H*×*W*×*C*^ is the input image, *M* ∈ *R*(0, 1)^*H*×*W*^ is the mask, and *I*_*out*_ ∈ *R*^*H*×*W*×*C*^ is the output image with information occlusion.

**Fig 3:**
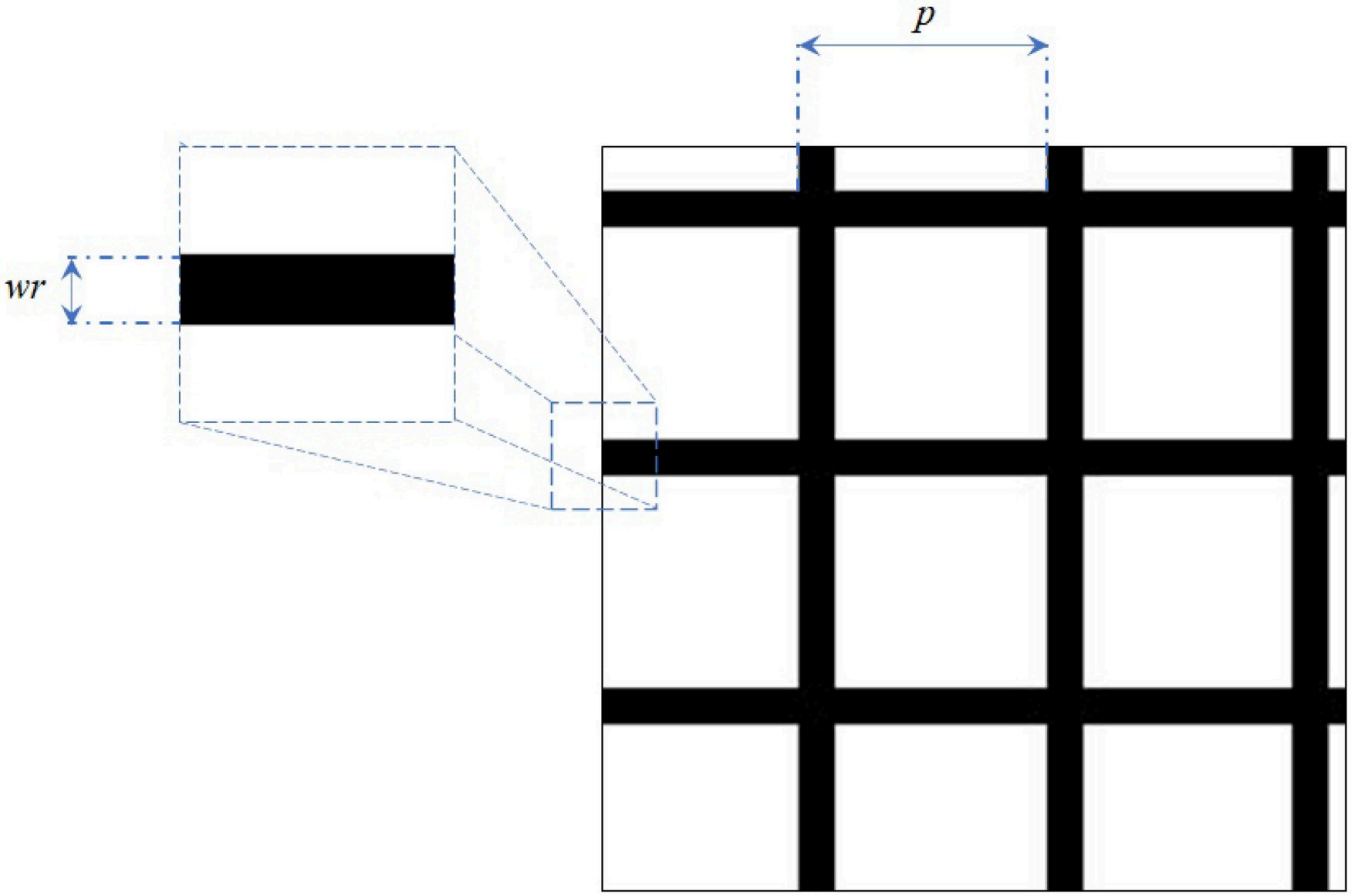


Fig 3 shows the mesh mask. For pixel(i,j) with a grey value of 1 in mask M, the corresponding regions of input I remain, but for pixel(i,j) with a grey value of 0, the corresponding regions are zeroed out. This operation should be applied after the image normalization operation.

Three parameters (p, wr and *m*_*prob*_) are introduced for controlling the shape of mask M, where p is the line-spacing, wr is the line width, and *m*_*prob*_ is the intervention rate of the overlaying operation. For p, some randomness is usually added to extend the applicability to a variety of images, which can be written as follows

$$\begin{array}{c} p=random(p_{max},p_{min}), \end{array}$$ (2)

For wr, it is a normalized ratio to the line spacing p in practice, which could be written as

$$\begin{array}{c} \text{wr}=pixels_{wr}/pixels_{p}, \end{array}$$ (3)

where *pixels*_*wr*_ and *pixels*_*p*_ are the number of pixels corresponding to the line spacing and the line width respectively.

To increase the randomness of segmentation, the entire mask is shifted by a random distance within a range of ±p/2 and rotated by a random angle within a range of 0° to 360°. Similar to the process reported in [13–16], the mask is superposed on the input image only in the training phase; in other words, the image without any data augmentation is put into the network for testing, as shown in Fig 2. Because the network has learned the representations distributed in multiple relevant parts, the segmentation operation is not required during testing.

## Experiment

To verify the validity and utility of our method, the experiments were performed with different computer vision tasks on the PyTorch platform. The performance evaluation standard for the image classification, object detection, and semantic segmentation tasks were top-1 accuracy, mAP, and mIoU, respectively, whose statistical approaches are described in [1, 5, 9, 10]. For demonstrating the performance difference, we selected some of the existing widely used state-of-the-art methods (e.g. Cutout, HaS, AutoAugment, and GridMask) for comparison in each task, all of whose results were cited from the corresponding original papers.

### Image classification

#### Imagenet

We ran our experiment on the ILSVRC-2012 dataset www.image-net.org to verify the performance of the proposed augmentation method. At the time of our experiments, the dataset had 1.4 million images: 1.2 million for training, 0.05 million for validation, and 0.15 million for testing. All of the images were on an average labelled with 1,000 categories for classification. For this dataset, we experimented with the official models of ResNet50 and ResNet101, which had been trained for 300 epochs with a batch size of 256. In the experiment, we used the common practice of baseline augmentation: resize a randomly cropped patch to the target size of 224 × 224 and horizontally flip it with a probability of 0.5.

During the training, we chose the hyperparameters of MeshCut as *p*_*max*_ = 224, *p*_*min*_ = 81, and wr = 0.14. The *m*_*prob*_ linearly increased from 0 to 0.8 with the training epochs increasing from 0 to 240 and then maintained this value to the end. The learning rate was determined by a MultiStepLR scheduler with an initial value of 0.1, which was reduced by 10% at the 100th, 200th, and 250th epochs. This proposed MeshCut method in our experiment was not accompanied by any other data augmentations, e.g. photometric distortion or information dropping. After all training epochs, the validation loss for the model trained with the MeshCut strategy can converge to a considerably smaller value at a relatively faster rate than that with the baseline augmentation, whereas its training loss is larger value than that with the baseline augmentation. Actually, this is an obvious signal that the MeshCut can enhance the learning difficulty, effectively forcing the network to learn more target features.

Because models after a longer training scheme are usually overfitted, to be fair, we selected the results with the highest validation accuracy in the entire training phase for comparison, as shown in Table 1. The comparison showed that the performance of the proposed MeshCut on the ILSVRC-2012 dataset improved ResNet50 by 1.8% (from 76.5% to 78.3%) and ResNet101 by 2.1% (from 77.8% to 79.9%), which outperformed the other listed augmentation methods. Another noteworthy aspect is the improved results of MeshCut after we incorporated it into AutoAugment, which achieved the state-of-the-art results on the two models.

**Table 1:** Experimental results of an image classification task on the ILSVRC-2012 dataset.

ILSVRC-2012	
*Model*	Accuracy (%)
*RseNet*50 [19]	76.5
+ *Cutout* [15]	77.1
+ *HaS* [16]	77.2
+ *AutoAugment* [24]	77.6
+ *GridMask* [13]	77.9
+ *MeshCut*	78.3
+ *MeshCut*+ *AutoAugment*	78.6
*RseNet*101 [20]	77.8
+ *GridMask* [13]	79.1
+ *MeshCut*	79.9
+ *MeshCut*+ *AutoAugment*	80.1

#### CIFAR-10

The CIFAR-10 dataset www.cs.toronto.edu/~kriz/cifar-10-python.tar.gz contained 50,000 images for training and 10,000 images for testing, all of which were labelled with 10 classes. We used the official ResNet-18 and WideResNet-28-10 models for the experiment. We set the hyperparameters *p*_*max*_, *p*_*min*_, and wr at 32, 10, and 0.15 respectively.

In term of the baseline augmentation, we first resized the input image to 40 × 40 and randomly cropped a patch with a size of 32 × 32. This experiment was then performed with the same training strategy and statistical method as those discussed in [15] and the Section of Imagenet: the results against other augmentation methods are presented in Table 2.

**Table 2:** Experimental results of the image classification task on the CIFAR-10 dataset.

CIFAR-10	
*Model*	Accuracy (%)
*RseNet*18 [4]	95.28
+ *Cutout* [15]	96.25
+ *HaS* [16]	96.10
+ *AutoAugment* [24]	96.07
+ *GridMask* [13]	96.54
+ *MeshCut*	96.79
+ *Cutout*+ *AutoAugment* [24]	96.51
+ *GridMask*+ *AutoAugment* [13]	96.64
+ *MeshCut*+ *AutoAugment*	96.87
*WideResNet* − 28 − 10 [25]	96.13
+ *Cutout* [15]	97.04
+ *HaS* [16]	96.94
+ *AutoAugment* [24]	97.01
+ *GridMask* [13]	97.24
+ *MeshCut*	97.52
+ *Cutout*+ *AutoAugment* [24]	97.39
+ *GridMask*+ *AutoAugment* [13]	97.48
+ *MeshCut*+ *AutoAugment*	97.64

The experimental results show that the proposed MeshCut strategy could improve upon the accuracies of all baseline models by a large margin and markedly surpassed all the listed information-dropping methods.

### Object detection

To determine the validity and the generalisation ability of MeshCut in an object detection task, we performed an experiment with Faster-RCNN-R50-FPN and Faster-RCNN-X101-FPN on the COCO 2017 dataset cocodataset.org. During the experiment, the models were initialised with the pre-trained weight of ImageNet and then fine-tuned on the COCO dataset. Photometric distortions, geometric transforms, normalised operation, and MeshCut were performed in this sequence. To determine the ability of MeshCut, we trained the models with the same hyperparameters as those described in [8], with 1×, 2×, and 4× training epochs. The other hyperparameters for MeshCut were the same as those given in the ImageNet section. The experimental results of MeshCut with various epochs are summarised in Table 3. In terms of the Faster-RCNN-R50-FPN, the results of mAP increased from 37.4% (baseline) to 38.6% (+1.2%) for 2× epochs, and from 37.4% (baseline) to 39.7% (+2.3%) for 4× epochs. For Faster-RCNN-X101-FPN, the results of mAP increased from 41.2% (baseline) to 42.9% (+1.7%) for 2× epochs. Compared with GridMask, our strategy could also achieve the best results in this task.

**Table 3:** Experimental results of the object detection task on the COCO dataset.

COCO2017			
*Model*	mAP (%)	AP50 (%)	AP75 (%)
*Faster* − *RCNN* − *R*50 − *FPN* [8]	37.4	58.7	40.5
+*gridmask*(2×) [13]	38.3	60.4	41.7
+*MeshCut*(2×)	38.6	60.5	41.9
+*gridmask*(4×) [13]	39.2	60.8	42.2
+*MeshCut*(4×)	39.7	61.2	42.5
*Faster* − *RCNN* − *X*101 − *FPN* [8]	41.3	63.5	44.4
+*gridmask*(2×) [13]	42.6	65.0	46.5
+*MeshCut*(2×)	42.9	65.3	46.9

### Semantic segmentation

To determine the capacity and the universality of MeshCut in a semantic segmentation task, we performed an experiment with the PSPNet model on the Cityscapes dataset [www.cityscapes-dataset.com]. We trained the model with the same hyperparameters as those suggested in [10], except for the longer (2×) training epochs. The above PSPNet model was also initialised with the pre-trained weights of ImageNet and then fine-tuned on the Cityscapes dataset www.cityscapes-dataset.com. The hyperparameters on MeshCut were the same as those given in the ImageNet section.

Although the mIoU of the PSPNet model could be improved significantly by introducing the GridMask, our MeshCut still achieved better results, as shown in Table 4.

**Table 4:** Experimental results of the semantic segmentation task on the Cityscapes dataset.

Cityscapes	
*Model*	mIoU (%)
*PSPNet*50 [10]	77.2
+ *gridmask* [13]	78.1
+ *MeshCut*	78.5
*PSPNet*101 [10]	78.3
+ *gridmask* [13]	79.0
+ *MeshCut*	79.3

## Discussion

In this section, we present an analysis to show the influences of various hyperparameters and the changes in the class activation mappings (CAMs) [15]. The related experiments were conducted using the same training and validation strategies as those discussed in the ImageNet and CIFAR-10 sections.

### Hyperparameter p

The line spacing p determines the size of the grid. To explore its impact, we selected ResNet18 to experiment with various ranges of p on CIFAR-10. The experiment results are presented in Table 5. For the same maximum bound (*p*_*max*_), the performance of MeshCut was better when p was distributed in a relatively larger interval, showing that a greater diversity of p could effectively improve the robustness of the network. In contrast, for the same distribution range, the performance became poor in the case of a small *p*_*min*_. The phenomenon that a smaller p was helpful to avoid segmentation failures did not seem to make sense, but it could be explained by the crash of feature extractions caused by the overfragmented segmentation. To effectively avoid this problem, we recommend a set of theoretical formulas as guideline to speed the hyperparameter tunning, as following:

$$\begin{array}{c} p_{\text{max}}=max\left(size\left(image\right)\right), \end{array}$$ (4)

$$\begin{array}{c} p_{\text{min}}=max\left(size\left(target\right)\right)/2, \end{array}$$ (5)

where the size(.) represents the statistic operator for the number of pixels in horizontal and vertical directions, the max(.) represents the operator for achieving the maximum value from the array. For the Imagenet and CIFAR-10, the majority of the target sizes are up to 60 90% of the image size, hence the optimal pmin in our experiment is within a range from 1/2 to 1/3 of image size.

**Table 5:** The results of various ranges of p.

Hyperparameter p	
*Range of p*	Accuracy (%)
[8, 24]	95.37
[10, 26]	96.48
[16, 32]	96.56
[13, 32]	96.67
[10, 32]	96.79

### Hyperparameter wr

**Fig 4:**
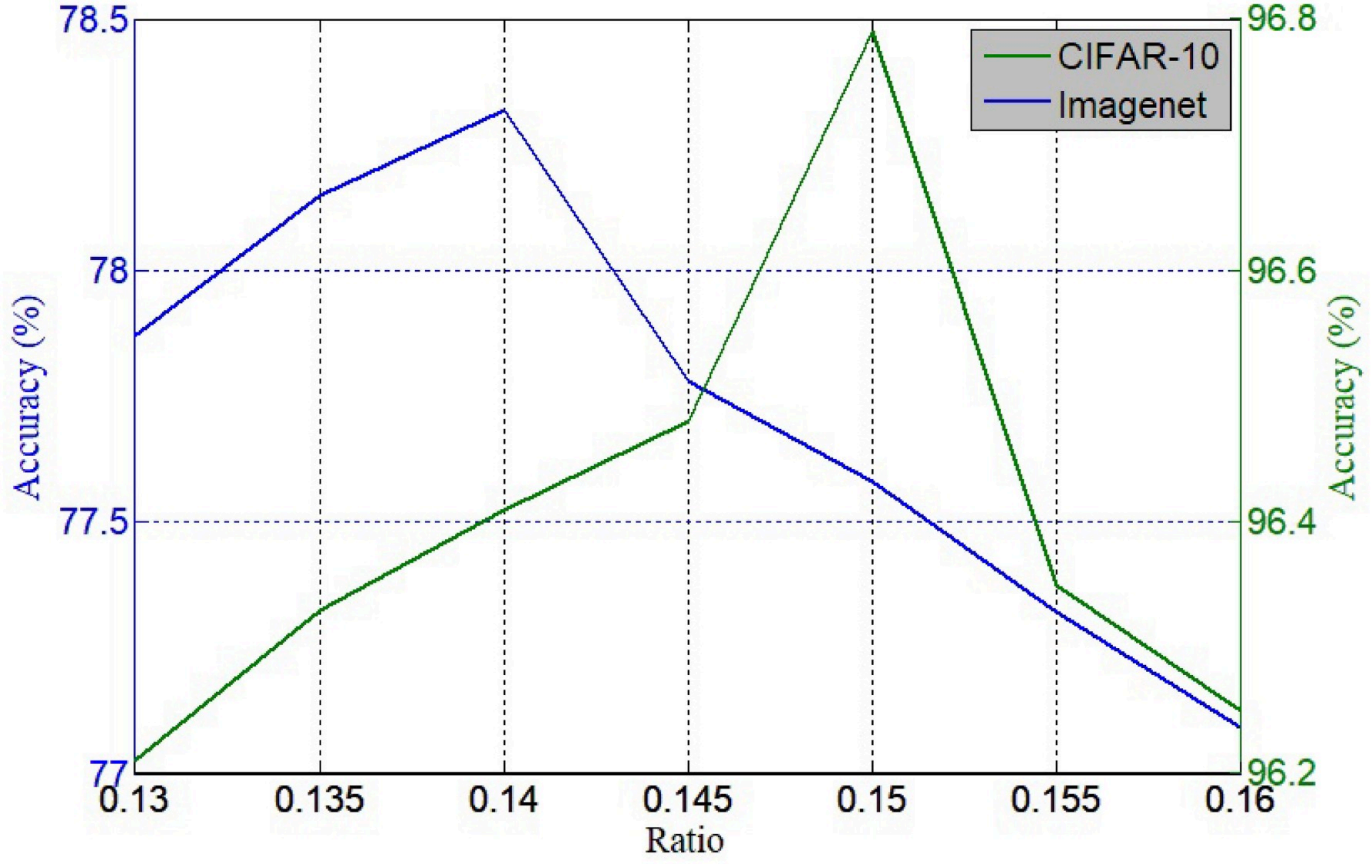


The hyperparameter wr determines the information retention ratio for an input image, which is important for the proposed algorithm to achieve good results, because artefacts may be unexpectedly introduced into a network in the case of a very small wr, whereas feature information may be excessively lost in the case of a very large wr. In practical use, wr is a constant, and its influence on the experimental results is shown in Fig 4.

Fig 4 shows the accuracy curve of ResNet50 trained on ImageNet. The figure shows that the performance of the proposed strategy was relatively poor in the case of a small wr. Under this condition, the capacity of MeshCut was limited, and the algorithm could not efficiently solve the overfitting. With an increase in wr, the accuracy improved and reached a maximum of 78.1% when the wr was at 0.14. Beyond this, there was a monotonic decrease in accuracy, meaning that underfitting began to appear. The same effect occurred on the curve of CIFAR-10 with ResNet18, except for the optimal value of wr, as shown in Fig 4. This very small difference made sense: more information needs to be reserved for perception in the case of a more complex dataset.

Actually, the wr determines the effective action depth of segmentation operation in the network, e.g. the larger segmentation gap still remains valid in a deeper layer after several down-sampling operations. Generally, the segmentation is expected to impact on the mid-level features, because the segmentation for the low-level features cannot efficiently implement the semantic segmentation while that for the high-level features may cause the problem of information loss. Herein, we also recommend a theoretical formula as guideline:

$$\begin{array}{c} \text{wr}=\left[n_{kernel}*size\left(image\right)/size\left(mid-level\right)\right]/p_{min}, \end{array}$$ (6)

where *n*_*kernel*_ is the size of convolution kernel, the size(mid-level) represents the input size of the down-sampling layer before the deepest layer on which the segmentation remains effect. For the Imagenet, the deepest effectively-segmented layer is selected the conv3-3 of the ResNet50 model, hence *n*_*kernel*_ = 3, size(mid-level) = 56. After calculation, the wr is within a range around 0.14. The same scheme can be applied for the CIFAR-10, and the similar results can be achieved.

### Scheme to use MeshCut

As for *m*_*prob*_, there exist two ways to apply MeshCut in practice: 1) training with a fixed *m*_*prob*_ and 2) training with increasing *m*_*prob*_. To determine a better-suited implementation, we selected ResNet18 to experiment on CIFAR-10 with two different schemes. For the first strategy, we set *m*_*prob*_ at 0.8 during the entire training phase. For the second strategy, we performed the experiment with the same strategy as that described in Section of CIFAR-10. The results showed that the second strategy led to a better result, as shown in Table 6.

**Table 6:** Results with two strategies for different values of *m*_*prob*_.

*m*_*prob*_	
*Model*	Accuracy (%)
*MeshCut*+ *fixed* *m*_*prob*_	96.43
*MeshCut*+ *increasing* *m*_*prob*_	96.79

### Loss curves

**Fig 5:**
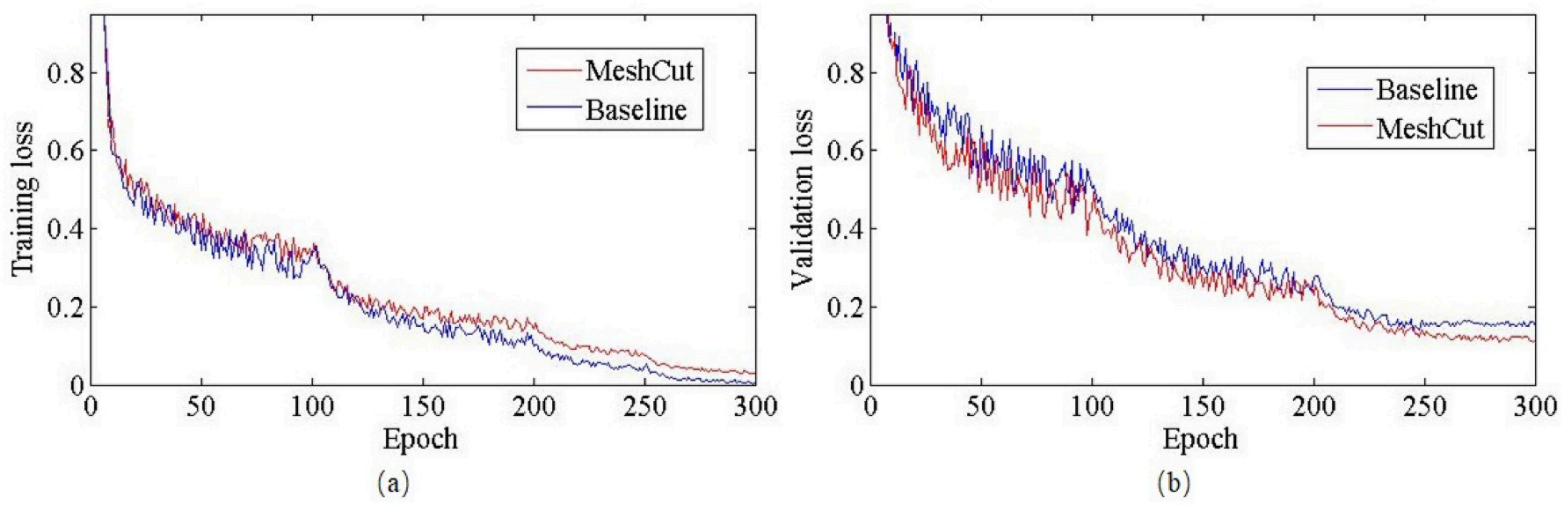


The loss curve is a critical indicator that reflects the affection of MeshCut on the training process. Considering this, we selected ResNet18 to experiment with baseline and MeshCut data augmentation schemes on CIFAR-10, whose loss curves in both training and validation phases are shown in Fig 5. Seen from it, the training loss for the model with baseline augment decreased to 0.002 after several epochs, while the validation loss only converged to 0.14, then the further decline became increasingly difficult. Comparing with this model, the training loss of model trained MeshCut strategy fell to 0.03 at a relatively slower rate, whereas its validation loss could dip below a much smaller value of 0.10. This is an obvious signal that the MeshCut could enhance the learning difficulty, effectively forcing the network to learn more target features.

### CAMs

**Fig 6:**
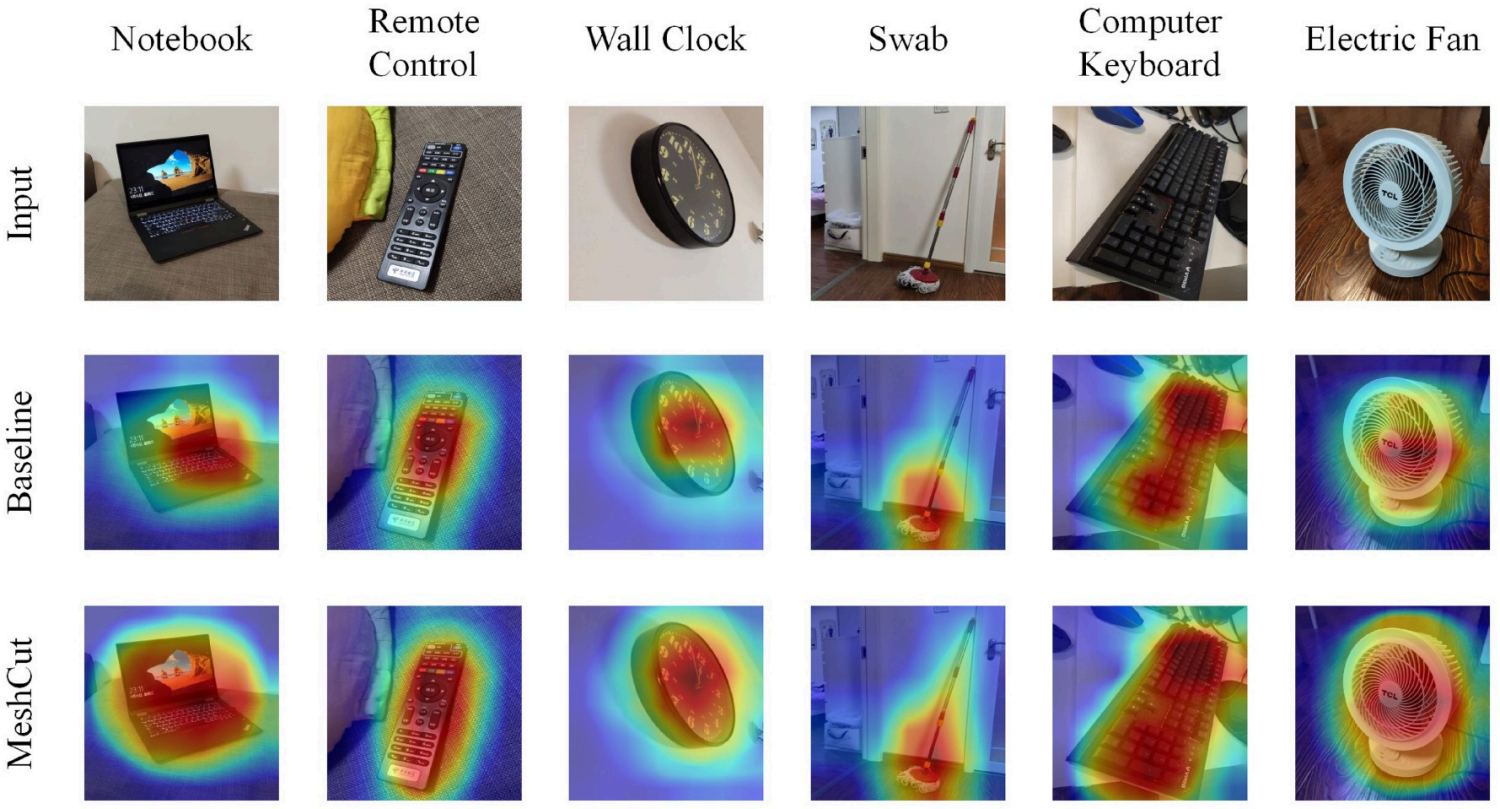


The key idea of MeshCut is to generate information occlusion with a mesh mask, with the purpose of forcing the network to learn more spatially distributed representations instead of focusing on only one discriminative feature. Therefore, the features learned by the network could directly respond to the efficiency of MeshCut. We introduced the CAMs for the conv5-3 of the ResNet50 model trained on ImageNet for the analysis. The comparison CAMs for various augmentation methods are shown in Fig 6. As a result, MeshCut trended to focus on larger spatially distributed regions, while the baseline augmentation tended to concentrate on a small region.

## Conclusion

We proposed a novel information-segregation-based augmentation strategy to solve the problem of overfitting for CNNs. By virtue of its mesh structure, the proposed MeshCut achieved state-of-the-art experimental results in various tasks and models. Extensive experiments analysed the influences of various hyperparameters on its performance. Moreover, the performance of such a strategy could be improved further by incorporation into other augmentation strategies, e.g. AutoAugment, which could make MeshCut a promising baseline strategy for data augmentation algorithms.
